# Budget impact analysis of the use of oral and intravenous therapy regimens for the treatment of relapsed or refractory multiple myeloma in Germany

**DOI:** 10.1007/s10198-020-01219-3

**Published:** 2020-07-11

**Authors:** Edin Basic, Mathias Kappel, Arpit Misra, Leopold Sellner, Boris A. Ratsch, Dennis A. Ostwald

**Affiliations:** 1 Takeda Pharma Vertrieb GmbH & Co. KG, Berlin, Germany; 2 Health Economics, WifOR, Darmstadt, Germany; 3 School of International Business and Entrepreneurship (SIBE), Steinbeis University Berlin, Berlin, Germany

**Keywords:** Budget impact analysis, Relapsed/refractory multiple myeloma, Progression-free survival, Partitioned survival analysis, Intravenous therapies, Oral therapies, I11

## Abstract

**Background:**

In Germany, several triplet therapies for treating relapsed or refractory multiple myeloma (rrMM) patients have recently been approved. While most of them are administered intravenously, ixazomib-based combination is the only orally bioavailable regimen.

**Objective:**

To conduct a 1-year and 3-year budget impact analysis (BIA) of different novel triplets to treat patients with rrMM in second or subsequent therapy lines accounting for costs covered by German statutory health insurance (SHI).

**Methods:**

A 3-state partitioned survival model (PSM) was developed to evaluate the budget impact of the following regimens: carfilzomib plus lenalidomide plus dexamethasone (KRd), elotuzumab plus lenalidomide plus dexamethasone (ERd), daratumumab plus lenalidomide plus dexamethasone (DRd), and ixazomib plus lenalidomide plus dexamethasone (IRd). The analysis included direct medical costs such as drug acquisition, comedication and preparation for parenteral solutions, drug administration and other 1-time costs, adverse event management costs and direct non-medical costs, such as transportation costs.

**Results:**

Based on current drug market shares in German healthcare market, the estimated costs after 1 year of treatment was €551 million (KRd), €163 million (ERd), €584 million (DRd), and €95 million (IRd). The total budget impact of €1393 million is mainly driven by drug acquisition and subsequent therapy costs.

**Conclusion:**

Among the regimens of interest, the oral-based therapy regimens offered cost advantages over intravenous-based therapy regimens. The higher overall costs of intravenous therapy regimens were attributed primarily to higher drug acquisition costs.

## Introduction

Multiple myeloma (MM) is an incurable, heterogeneous blood cancer with serious disease-related complications accounting for 1% of all cancer diagnoses worldwide and 13% of all hematologic malignancies [1]. In Germany, there are around 6500 new cases of MM each year with the median age at onset of 71 years for men and 74 years for women [2]. During the course of the disease, nearly all patients with MM relapse or become refractory to the upfront therapy. More recently, several new agents, such as the proteasome inhibitors (PIs) carfilzomib and ixazomib, and two monoclonal antibodies, elotuzumab and daratumumab, have been approved for the treatment of MM patients who received at least one prior line of therapy. These new agents, given in combination with corticosteroids and immunomodulatory drugs, in particular lenalidomide, have demonstrated to be efficacious in extending progression-free survival (PFS) and time to progression [3–6]. While the majority of these new drugs are only administered parenterally (specifically intravenously), the ixazomib-based combination is the only orally bioavailable regimen. Hence, the oral formulation of ixazomib makes it unique in the sense that it is an integral part of the only currently approved oral triplet for relapsed or refractory MM (rrMM) that incorporates both a PI and an immunomodulatory agent. It has been postulated that oral administration of anticancer drugs may contribute to improving patients’ quality of life (QoL), since oral administration avoids the inconvenience of infusions, the risk of infusion-related infections or extravasations, stress related to infusion, and the need for additional administration visits [7, 8]. Moreover, the oral and the intravenous administration of anticancer drugs may place different financial burden on the healthcare system, not only as a result of differences in drug acquisition costs, but also the differences in the reimbursement of additional services associated with oral and intravenous administration, respectively. For example, in Germany, the physician fees for administration of intravenous therapies are twice as high as for oral therapies and there is an extra reimbursement fee for additional time spent administering intravenous therapies. In addition, travel costs for administration of intravenous therapies are routinely covered by the German statutory health insurance (SHI). These differences may create disincentives in the use of oral therapies in rrMM patients within the German healthcare system. However, knowledge of the total costs associated with the oral and parenteral drug use for treatment of rrMM in Germany is scarce. Therefore, the aim of the present study was to conduct a budget impact analysis of the recently approved therapy regimens used in the treatment of rrMM patients accounting for costs covered by the German SHI.

## Methods

### Model structure

**Fig. 1:**
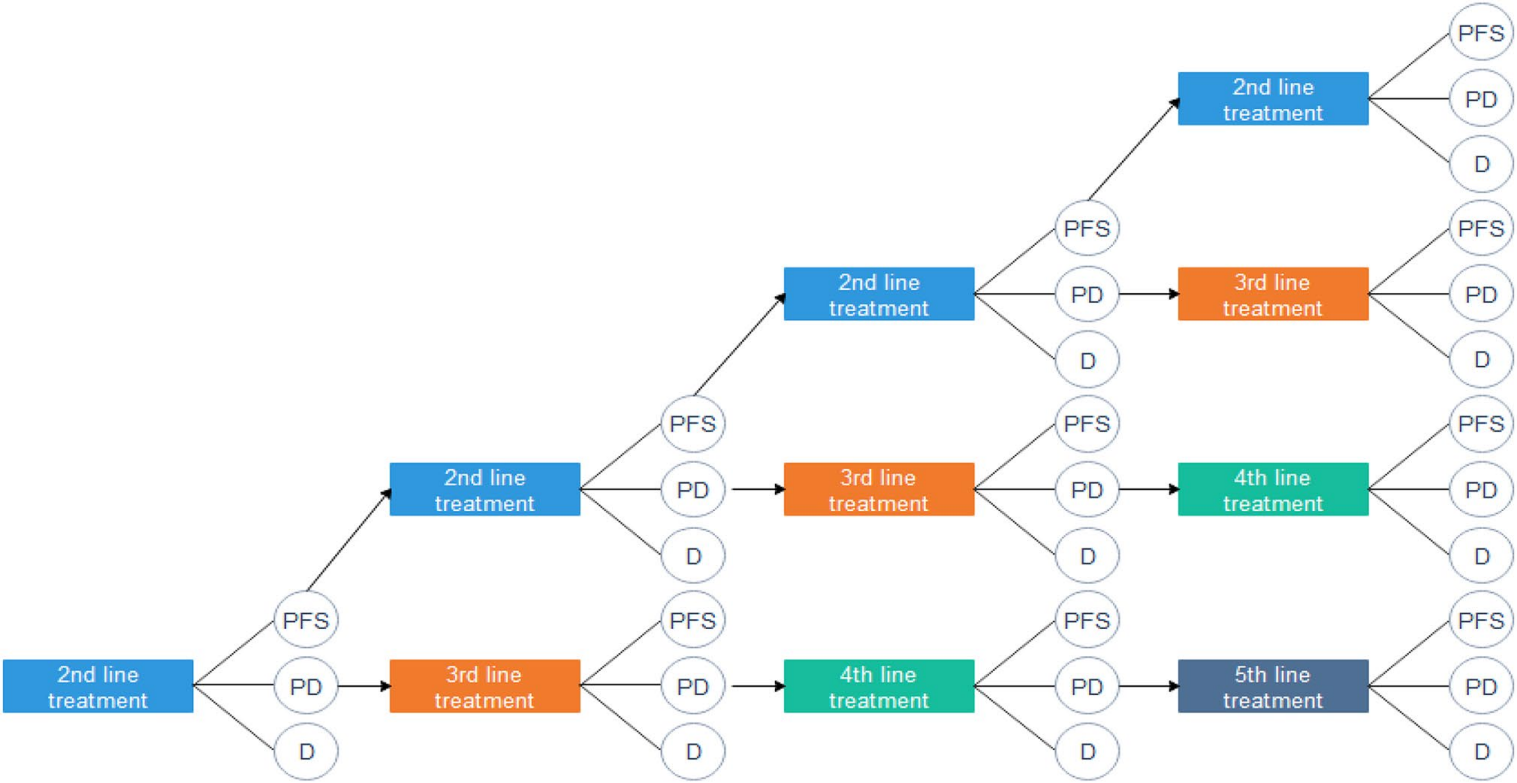
Patient pathway in budget impact model

To perform the budget impact analysis (BIA), a three-state partitioned survival model (PSM) was used, which classifies patients into states of PFS, progressive disease (PD), and death (D). All patients were assumed to have started in the PFS state and either remained progression free (PF), progressed, or died in subsequent cycles. Time to progression was derived from the difference between the areas under the PFS and overall survival (OS) curves. After disease progression, up to three subsequent therapy lines were considered (Fig. 1). To reflect the dosing schedules for the included drug regimens, a 28-day cycle was used in the model. Treatment duration was based on the median PFS observed in the clinical trials.

The budget impact analysis was performed from the perspective of the German SHI over a 1-year and 3-year time horizon. A dynamic cohort model to account for the prevalent cohort in the starting year 2019 as well as the incident cohorts in the following years was applied. Additionally, the yearly cohort size was divided by 13 (equal to the number of 28-day cycles per year) to reflect a constant patient-inflow into the model.

The budget impact analysis was conducted using Microsoft Excel 2016 (Microsoft Coporation).

### Regimens of interest

The focus of the current analysis was to evaluate the four triplet therapy regimens recently approved for the treatment of rrMM. These were carfilzomib plus lenalidomide plus dexamethasone (KRd), elotuzumab plus lenalidomide plus dexamethasone (ERd), daratumumab plus lenalidomide plus dexamethasone (DRd), which were referred to as intravenous therapy regimens. In addition, ixazomib plus lenalidomide plus dexamethasone (IRd), referred to as oral therapy regimen, was considered. Lenalidomide in these regimens is normally given until progression. Patients progressing under treatment with these triplet regimens are considered to be lenalidomide refractory. Therefore, lenalidomide-free regimens are preferred after lenalidomide-based treatment [9]. Hence, three lenalidomide-free therapy regimens were considered after the progression on the four initial triplets mentioned above. These additional three therapy regimens were pomalidomide plus dexamethasone (Pd), daratumumab plus bortezomib plus dexamethasone (DVd), and panobinostat plus bortezomib plus dexamethasone (FVd).

### Patient pathway

The analysis was limited to the listed therapies above as well as to the order and frequency in which the therapies were prescribed. It was assumed that patients start treatment on one of the four lenalidomide-based therapies in second line, namely, KRd, ERd, DRd or IRd. Once those patients experienced a disease progression, lenalidomide-free therapies (Pd, DVd or FVd) were prescribed in third treatment line, only. Patients with a repeated disease progression were eligible for any of the considered therapies in fourth and fifth treatment line, excepting those previously received. This setting allowed to reflect 312 different treatment combinations across the four therapy lines.

### Patient population

The baseline target population of the model was defined as all adult patients with MM who have received at least one prior therapy and who may initiate a second-line therapy and more. The determination of the prevalent target population was based on the estimates from the German Centre for Cancer Registry Data (ZfKD) which regularly reports data on incidence and prevalence of cancer in Germany for the entire population as well as for population stratified by age and gender [10]. As the ZfKD only reports data for the overall multiple myeloma population, additional criteria were applied to extract the relevant data on rrMM patients [11, 12]. For the derivation of the incidence population, the same approach as for the baseline target population was applied. These resulted in a prevalent target population of 10,262 in 2019. The incident populations in 2020 and 2021 were estimated at 2337 and 2417 patients, respectively. The distribution of the prevalent target population among the considered therapy regimens was based on the results from a nationwide, multi-institutional survey on treatment of multiple myeloma patients in Germany (TherapyMonitor) [13]. Previous analyses revealed that the database from the TherapyMonitor has a good external validity to the German population regarding the distribution of treated patients with multiple myeloma [14].

### Model parameters

#### Clinical efficacy data

Clinical efficacy data in terms of PFS and OS curves for each regimen of interest were retrieved from clinical trials [3–6, 15–17]. ERd, DVd and IRd data on OS curves were obtained from the EMA’s assessment reports [18–20]. Since the observed survival distributions for PFS and OS were limited by the time of follow-up in published sources, it was necessary to extrapolate them over a lifetime horizon. This was achieved by extracting individual data points from the published Kaplan–Meier (KM) curves for OS and PFS using the WebPlotDigitizer developed by Rohatgi [21]. In addition, the number of patients at risk for each arm at regular time intervals during the follow-up was extracted. This information, usually known as the risk table, was presented beneath the published KM curves. By incorporating the information provided in the risk table, the accuracy of the approximated time-to-event data was improved [22]. Then the extracted data was reconstructed using an algorithm (ipdfc) developed by Wei and Royston [23] for use in STATA. The algorithm estimates the number of censoring, the number of events, the censoring time, and event time. In addition, it corrects also for monotonicity violators, a situation where a pair of adjacent times and corresponding survival probabilities is inappropriately ordered. Table 1 shows the obtained outputs for treatment and control arms, respectively.

**Table 1:** Comparison of the results of the PFS Kaplan–Meier curves from respective trials to those reconstructed following the use of the algorithm in STATA

	Number of events				Median PFS				Hazard ratios (95% CI)	
	Control		Treated		Control		Treated			
Therapy regimen	O	R	O	R	O	R	O	R	O	R
KRd	224	225	207	209	17.6	19.4	26.3	26.7	0.69 (0.57–0.83)	0.71 (0.59–0.86)
ERd	205	201	179	177	14.9	14.9	19.4	19.7	0.70 (0.57–0.86)	0.70 (0.57–0.86)
DRd	116	112	53	52	18.4	16.8	NR	38.4	0.37 (0.27–0.52)	0.39 (0.28–0.54)
IRd	157	156	129	127	14.7	14.9	20.6	20.8	0.74 (0.59–0.94)	0.74 (0.59–0.94)
Pd	133	133	233	235	1.9	2.4	4.0	4.6	0.48 (0.39–0.60)	0.48 (0.39–0.60)
DVd	NR	58	NR	40	6.5	6.6	9.3	10.0	0.52 (0.33–0.81)	0.52 (0.34–0.78)
FVd	NR	261	NR	209	8.0	8.4	12.0	12.1	0.63 (0.52–0.76)	0.66 (0.54–0.79)

*O* observed, *R* reconstructed, *CI* confidence interval

The reconstructed PFS and OS curves, respectively were then fitted to a variety of common parametric distributions, using the maximum likelihood methodology. The distributions that were tested included the exponential, Weibull, Gompertz, log-normal, and log-logistic. The final distributions were chosen based on the following criteria: (1) comparison of Akaike and Bayesian information criteria (AIC/BIC); (2) clinically plausible long-term projections; (3) a comparison of predicted median PFS time and the published figures; (4) visual inspection of the fit to the observed data over the available follow-up time; and (5) residuals against a 45° line (Cox-Snell residual analysis). For PFS, a Gompertz distribution was selected for KRd, DVd, and FVd; a Weibull accelerated failure time (AFT) distribution for ERd, DRd, and Pd; and a log-normal distribution for IRd. For OS, a Gompertz distribution was selected for IRd, KRd, ERd, and FVd; a Weibull distribution for DVd; and a Weibull AFT distribution for DRd and Pd.

#### Costs

The analysis included both direct medical costs and direct non-medical costs (i.e., transportation costs) which are covered by the German SHI. The direct medical costs included the initial and subsequent-line drug acquisition costs, comedication costs, drug administration costs including administration, patient monitoring and laboratory tests, as well as costs for management of adverse events (AE). Drug administration time, dosing, clinical examinations before treatment initiation, and comedications were based on the prescribing information for each agent [24–29]. The calculation of drug dosing was based on the relative dose intensity per mean body surface area (BSA) or mean body weight (BW) for carfilzomib and bortezomib or dexamethasone and elotuzumab, respectively. According to the German microcensus, an average German has a BSA of 1.89 m^2^ and a BW of 76.3 kg. Thus, the estimated drug costs correspond to the number of drug packages/vials used to meet the previously calculated dose intensity. Doses and prices per package were retrieved from a public price list (Lauer-Taxe) [30]. The drug administration costs were retrieved from the physicians’ fee schedule (Einheitlicher Bewertungsmaßstab, EBM).

The incidence of AEs associated with each therapy regimen was obtained from pivotal trials, and the costs of AEs were obtained from the morbidity-oriented risk structure compensation scheme (Morbi-RSA) in Germany [31]. AEs of grade 3 or higher, which were consistently defined across clinical trials, occurred in at least 5% patients, and their costs were listed in the Morbi-RSA only were considered for all regimens. These criteria resulted in 2 AEs that were included in the model: anemia and neutropenia. For each AE, the incidence rate per cycle was multiplied by the respective cost to obtain the AE-associated costs.

The considered non-medical costs included transportation costs, which are routinely covered by SHI for oncological intravenous treatments. These costs commonly refer to roundtrips per drug administration and were estimated according to the mode of transport and distance travelled. The average distance travelled for an intravenous rrMM treatment is 37.2 km in Germany [32]. Due to the poor health condition of rrMM patients, it was assumed that half of the patients used a private mode of transportation driven by a relative and the other half by taxi.

All costs were adjusted for a cycle length of 28 days. Costs were not discounted as recommended by the International Society for Pharmacoeconomics and Outcomes Research (ISPOR) Task Force for BIAs [33]. Data are presented as rounded numbers, although model calculations were performed without rounding. The cycle length and regimen dosing in combination with the drug acquisition costs per cycle as well as other considered cost components are shown in Table 2.

**Table 2:** Description of drug administration time, dosing, and cost categories for each therapy regimen

	KRd	ERd	DRd	IRd		Pd	DVd	FVd
Drug cost per cycle								
Dose	K: 20 and 27 mg/m^2^ R: 25 mg d: 40 mg	E: 10 mg/kg R: 25 mg d: 28 and 40 mg	D: 16 mg/kg R: 25 mg d: 40 mg	I: 4 mg R: 25 mg d: 40 mg	P: 4 mg d: 20 and 40 mg		D: 16 mg/kg V: 1.3 mg/m^2^ d: 20 mg	V: 1.3 mg/m^2^ F: 20 mg d: 20 mg
Administrations per cycle	K 20 mg/m^2^: 2 (cycle 1) K 27 mg/m^2^: 4 (cycle 1), 6 (cycles 2–12), 4 (cycles 13–18) R: 21 (all cycles) d: 4 (all cycles)	E: 4 (cycles 1–2), 2 (cycles 3+) R: 21 (all cycles) d 28 mg: 4 (cycles 1–2), 2 (cycles 3+) d 40 mg: 2 (cycles 3+)	D: 4 (cycles 1–2), 2 (cycles 3–6), and 1 (cycles 7+) R: 21 (all cycles) d: 4 (all cycles)	I: 3 (all cycles) R: 21 (all cycles) d: 4 (all cycles)	P: 21 (all cycles) d 20 mg: 4 (all cycles, age < = 75) d 40 mg: 4 (all cycles, age > 75)		D: 3 (cycles 1–3), 1 (cycles 4+) V: 4 (cycles 1–8) d: 8 (cycles 1–8)	V (age ≤ 75): 4 (cycles 1–8), 2(cycles 9–17) F: 6 (all cycles) d (age ≤ 75): 8 (cycles 1–8), 4 (cycles 9–17)
Cycle length	28 days	28 days	28 days	28 days	28 days		21 days (cycles 1–8) 28 days (cycles 9+)	21 days
Costs of therapy per cycle^a^	Cycle 1: €14,071 Cycles 2–12: €14,850 Cycles 13+ : €12,439	Cycles 1–2: €19,372 Cycles 3+ : €13,495	Cycles 1–2: €37,650 Cycles 3–6: €22,634 Cycles 7+ : €15,126	All cycles: €13,683	All cycles: €9119		Cycles 1–3: €28,697 Cycles 4–8: €13,682 Cycles 9+ : €7508	Cycles 1–8: €10,565 Cycles 9–17: €7479 Cycles 18+ : €0
Costs for management of AE per cycle	All cycles: €76	All cycles: €103	All cycles: €152	All cycles: €86	All cycles: €389		All cycles: €324	All cycles: €102
Transportation costs per cycle	Cycles 1–12: €183 Cycles 13+ : €122	Cycles 1–2: €122 Cycles 3+ : €61	Cycles 1–2: €122 Cycles 3–6: €61 Cycles 7+ : €31	All cycles: €0	All cycles: €0		Cycles 1–8: €122 Cycles 9+ : €31	Cycles 1–8: €122 Cycles 9–17: €61 Cycles 18+ : €0
Administration costs per cycle and other 1-time costs	All cycles: €110	All cycles: €125	Cycles 1: €170 Cycles 2+ : €139	All cycles: €77	All cycles: €72		Cycles 1–8: €228 Cycles 9–13: €261 Cycles 14+ : €140	Cycles 1–17: €93 Cycles 18+ : €0
Comedication and preparation costs for perenteral solution	Cycles 1–12: €486 Cycles 13+: €324	Cycles 1–2: €599 Cycles 3+: €299	Cycles 1–2: €745 Cycles 3–6: €372 Cycles 7+: €186€	All cycles: €0	All cycles: €0		Cycles 1–3: €883 Cycles 4–8: €510 Cycles 9+ : €186	Cycles 1–8: €324 Cycles 9–17: €162 Cycles 18+ : €0

*K* carfilzomib, *R* lenalidomid, *d* dexamethasone, *E* elotzumab, *D* daratumumab, *I* ixazomib, *P* pomalidomide, *V* bortezomib, *F* panobinostat

^a^The cost per cycle was determined using unit sizes listed in the Lauer Taxe [30]. Unit sizes were selected to minimize vial waste, and it was assumed that no vial sharing was allowed

### Modeling scenarios

To estimate the budget impact of rrMM patients starting second-line treatment with KRd, ERd, DRd or IRd, 6 different scenarios were considered within a 1-year and 3-year time horizon.

#### 1-year budget impact

Using estimates from the TherapyMonitor Report, the current market share of the four considered therapy regimens was 20%, 14%, 5%, and 4% for KRd, DRd, ERd, and IRd, respectively, in Germany during the 1st quarter of 2019 [13]. To allow for a meaningful comparison of budget impact between the four regimens of interest, these market shares were reweighted to sum up to 100% and assumed to hold true for the entire year 2019. Hence, the reweighted market shares in the reference scenario were estimated at 46%, 32%, 13% and 9% for KRd, DRd, ERd and IRd, respectively. The budget impact for the reference scenario was compared to several new market shares/penetration scenarios. These included the equivalence scenario, where all four triplets had an equal market share (25% each); the KRd-Scenario, where the market share of KRd was assumed at 100% and 0% for all other triplets; the DRd-Scenario, where the market share of DRd was assumed at 100% and 0% for all other triplets; the ERd-Scenario, where the market share of ERd was assumed at 100% and 0% for all other triplets, and the IRd-Scenario, where the market share of IRd was assumed at 100% and 0% for all other triplets. The flow of treatment of the progressed patients after the second line is described in Table 3. The market shares of Pd, DVd, FVd for the third treatment line were derived from the TherapyMonitor and reweighted to sum up to 100%. Once a patient progresses from the third line to the subsequent lines, that patient cannot receive the medication received in the second and third therapy line. For example, if a patient received KRd in the second line and Pd in the third line, this patient cannot receive KRd or Pd in the fourth line and the fifth line. To account for this assumption, patients not eligible for a particular therapy are redistributed among the remaining therapies in the fourth and fifth lines.

**Table 3:** Description of treatment after progression on the initial therapy

	KRd	ERd	DRd	IRd	Pd	DVd	FVd
2nd line	Varying market shares according to selected scenario						
3rd line^a^					36%	54%	10%
4^th^ line^a^	10%	4%	11%	19%	20%	30%	6%
5^th^ line^a^	10%	4%	11%	19%	20%	30%	6%

^a^Market shares vary according to the previous individual treatment pathway

#### 3-year budget impact

In all 3-year scenarios, the prevalent cohort (2019) in second line reflects the market share observed in the TherapyMonitor Report during the 1st quarter 2019. The treatment of the progressed patients after the second line followed a similar logic that was applied in the 1-year analysis (Table 4). The incident cohorts (2020 and 2021) in the second line vary with the chosen scenario and reflect the projected market shares in subsequent years. In reference scenario, the market share of KRd and ERd decreased from 46 to 18% and from 13 to 8% (2019–2021), respectively, and the market shares of DRd and IRd increased from 32 to 63% and from 9 to 11%, respectively. This scenario reflects a strong market penetration of DRd that was observed in 2018 [13]. As the IRd was associated with the most-favorable safety profiles among the considered therapy regimens, a small uptake was assumed, especially due to the increased use in patients at advanced age affected by comorbid conditions. The strong decrease of KRd therapy regimen was related to the frequently reported cardiotoxicty problems in patients treated with KRd and decrease in market share observed during the previous year [13, 34]. In scenarios with 100% market share for each therapy regimen, it was assumed that in 2020 and 2021 the market is dominated by a specific therapy regimen. One exception is the scenario with equal market shares, where equal market shares were assigned to all drugs in the second and subsequent treatment lines.

**Table 4:** Budget impact results by cost category and over 1-year and 3-year time horizon in Euro

Scenario	Drug acquisition costs	Administration and other 1-time costs	Comedication and preparation costs for parental solution	Adverse event costs	Transportation costs	Subsequent line costs^a^	Total costs	Difference^b^
1-year time horizon								
Reference	1243	8	29	0.6	8	104	1393	-
Equivalence	1196	8	23	0.6	6	95	1317	5.5% (76)
IRd	961	5	0	0.5	0	99	1060	23.9% (333)
KRd	1025	8	35	0.4	13	116	1197	14.1% (196)
DRd	1720	10	29	0.8	5	59	1824	30.9% (431)
ERd	1042	9	26	0.5	5	174	1257	9.8% (136)
3-year time horizon								
Reference	5955	47	120	3	34	2187	8346	–
Equivalence	5783	44	96	3	25	2080	8032	3.8% (314)
IRd	5721	44	99	3	29	2241	8119	2.7% (227)
KRd	5747	46	127	3	39	2218	8181	2.0% (165)
DRd	6151	48	119	3	32	2129	8483	1.6% (137)
ERd	5757	47	120	3	33	2312	8273	0.9% (73)

^a^In the second-line, costs were differentiated by drug acquisition costs, comedication and preparation costs for parenteral solutions, drug administration and other 1-time costs, transportation costs and AE management costs. Subsequent therapy costs were defined as the sum of the listed cost components in second-line across third, fourth and fifth therapy line

^b^Difference between different scenarios and the Reference scenario. Below the proportion as well as absolute numbers (in brackets) are displayed

### Sensitivity analysis

The sensitivity of the model was assessed through a combination of one-way and scenario analyses for equivalence scenario and 1-year time horizon. First, deterministic sensitivity analyses were conducted to identify the most influential inputs on the total budget. Second, for scenario analyses, the most plausible alternative values were used. Scenario analyses performed included: (a) alternative body surface area (1.8–1.89 m^2^); (b) alternative body weight (70–76.3 kg); (c) replace the treatment duration based on median PFS by separately calculated duration of treatment (DoT) curves; (d) use an alternative parametric distribution such as the second-best fit curve; and (e) assume a 100% mortality rate on progression and thus, only consider second-line treatment costs.

## Results

### 1-year time horizon

Table 4 presents the budget impact for the prespecified scenarios and the contribution of the individual cost components to the annual total costs. Predicted total costs over 1 year with the current market share (reference scenario) were €1393 million, of which 89.2% were related to drug acquisition costs, 7.5% to costs associated with subsequent lines, 2.1% of the total costs were generated by comedications and preparation costs for parenteral solutions, whereas the transportation as well as administration and other one-time costs accounted for 0.6% of the total costs, respectively. In the scenario with equal market shares of the four regimens of interest (equivalence scenario), predicted total costs over 1 year were €1317 million, i.e., lower than in reference scenario by 5.5% (€76 million). These savings were mostly driven by lower drug acquisition costs (€47 million), followed by lower costs for subsequent lines (€9 million) and comedications and preparation costs for parenteral solution (€6 million). The use of oral therapy alone (IRd-Scenario) resulted in 23.9% (€333 million) lower total costs compared to the reference scenario with the largest cost reduction achieved among drug acquisition costs (€282 million) and comedications and preparation costs for parenteral solutions (€29 million). For scenarios with the use of intravenous therapies alone (KRd-Scenario, DRd-Scenario, and ERd-Scenario), somewhat different results were observed. Whereas the use of KRd and ERd alone resulted in total cost reductions of 14.1% and 9.8%, respectively, compared to reference scenario, the total costs increased for DRd alone by 30.9%. The key driver of cost reduction for scenarios with the KRd and ERd use alone were savings in drug acquisition costs. However, the costs associated with subsequent lines of therapy increased in both scenarios. The relatively large increase in the total costs for the DRd alone scenario was mainly driven by the drug acquisition costs which increased by 38.4% (€477 million). On the other hand, similar relative reduction of costs associated with subsequent lines as well as transportation costs was observed. However, due to the relatively low impact of all cost components other than the drug acquisition costs (drug acquisition costs represents primary cost component across all scenarios, ranging from 83 to 94%) on the total costs, these cost reductions could not offset the increase in drug acquisition costs.

### 3-year time horizon

Predicted total costs over 3 years for the reference scenario were €8346 million, of which 71.3% were related to drug acquisition costs, 26.2% to costs associated with subsequent therapy lines, comedications and preparation costs for parenteral solutions generated 1.4% of the total costs, and administration and other one-time costs as well as transportation costs accounted for 0.6% and 0.4% of the total costs, respectively (Table 4). The scenario with equal market shares resulted in total costs of €8032 million. The savings of €314 million compared to the reference scenario were mainly driven by lower expenses for drug acquisition costs and reduction in costs associated with subsequent lines and comedications and preparation costs for parenteral solutions. The use of oral therapy alone (IRd-Scenario) in the years 2020 and 2021 generated savings of €227 million compared to the reference scenario. Saving effects were observed for all cost components except costs associated with subsequent therapy lines. The scenarios with the use of KRd as well as ERd alone yielded savings compared to the reference scenario; however, the magnitude of savings was lower than those for the IRd-Scenario (€165 and €73 million, respectively). The only scenario that generated higher total costs compared to the reference scenario was the use of DRd alone in the years 2020 and 2021. Compared to the reference scenarios the total costs increased by €137 million. The key driver of the increased total costs were the drug acquisition costs.

### Sensitivity analyses

**Fig. 2:**
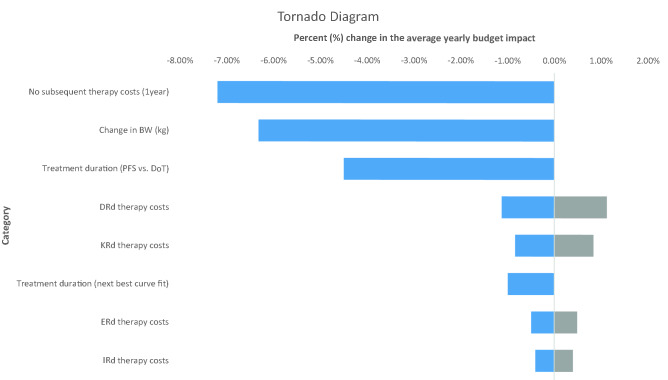
Tornado diagram illustrating results of deterministic sensitivity analyses

Figure 2 indicates that the model and the four starting triplets are most sensitive to the exclusion of the subsequent treatment lines. The total budget impact is reduced by 7.2% down to €1222 million as a result of only considering second-line treatment costs. A variation in BW or BSA corresponds to a variation in dose intensity for all triplets, except IRd. However, the varied dose intensity leads to a reduction of needed drug packages for DRd, only. As expected, since DRd makes up a relatively larger proportion of the total budget impact, the decline from 4 to 3 drug packages per DRd-treatment day is the second largest driver of costs on the total budget. By estimating the budget impact on DoT curves rather than median PFS, the total costs decrease by 4.5%. However, the relative impacts between the two scenarios are consistent. Varying the drug acquisition costs by 5% affect the triplet therapy costs moderately (0.4% for IRd to 1.12% for DRd). Using second-best fit curves, the impact on total costs (reduction by 0.99%) is almost negligible. The second-best curve for IRd is relatively more conservative than the best fit (67% PFS vs. 60% PFS by end of 1 year). For KRd, ERd and DRd, the second-best curve results in more patients in PFS by end of 1 year, and therefore, these drugs accumulate relatively less costs in subsequent treatment lines.

## Discussion

In Germany, all medical costs related to cancer care are covered in full by the German healthcare system. In principle, there is no substantial out-of-pocket cost that may prevent patients from accessing cancer care. Nonetheless, the present analysis demonstrated that the use of oral-based therapy regimens for treatment of rrMM patients may lead to savings for the German SHI as a consequence of both reduced direct costs and direct non-medical costs. This cost advantage of using oral-based therapy regimens compared to intravenous therapy regimens was especially pertinent to a 1-year time horizon and was primarily driven by the reduction of the acquisition costs of the agent itself. Other notable relative differences between oral-based and intravenous-based therapy regimens were observed for all other cost components but AE costs. The lower administration costs as well as transportation costs associated with oral-based therapy regimen can be explained by a lower burden for administration visits. Although these cost components are a relatively small percentage of the total cost, such costs are clearly associated with patients' health-related QoL potentially affecting patients’ ability to remain on long-term treatment [35, 36]. The benefit of using oral-based therapy regimen became further evident by examining the preparation costs for parenteral solutions, being the third most important cost component of the total costs. For instance, a recently published report by Germany’s second largest sickness fund reported that these costs account for about 10% of all pharmaceutical costs and more than 90% of these costs are associated with parenteral solutions for monoclonal antibodies and cytostatic parenteral solutions [37]. The costs of AE management were similar and relatively low across all triplet therapies. However, this is in contrast with recently published data which reported that oral-based therapy is associated with relatively lower costs of AE management due to their better safety profiles [38, 39]. In this study, a number of additional AEs were not taken into account as it was not possible to obtain costs from Morbi-RSA or the definitions differed across the clinical trials. For example, the occurrence of cardiovascular events has been frequently reported for the carfilzomib-based combinations, whereas a higher likelihood for occurrence of severe infections was observed for the daratumumab-based combinations [6, 34]. Hence, the current analysis likely underestimates the AE management costs for German SHI due to rrMM. In terms of the costs for subsequent treatment lines, somewhat different results between the triplet therapies were observed. As expected, for both time horizons, the lowest costs for subsequent therapy lines were accumulated in the DRd alone scenario. Among all considered therapies, DRd had the highest estimated PFS resulting in the lowest number of patients progressing to subsequent lines [40].

With respect to the impact of the drug acquisition costs on the total costs, slightly different results were observed for the two time horizons. The drug acquisition costs accounted for about 90% of the total costs for the 1-year time horizon; however, this dropped to about 70% in the 3-year time horizon. This is not surprising given that there are more patients in the disease progressed stage or fewer patients survived the 3-year time horizon compared to the 1-year time horizon, which may impact the accumulation of drug acquisition costs. Furthermore, the relative differences in drug acquisition costs between the scenarios varied according to the chosen time horizon with minor differences in the 3-year time horizon compared to a 1-year time horizon. This is directly related to the reduced costs for the intravenous-based therapy regimens after first year of treatment due to dose intensity reductions. Hence, from the second treatment year, the drug acquisition costs were lowest for KRd therapy regimen followed by ERd, IRd, and DRd.

The presented cost trends for German SHI are also in agreement with the recently published data from other countries comparing costs of oral-based and intravenous-based therapy regimens for treatment of rrMM [37, 38]. For instance, Ailawadhi and colleagues compared direct and indirect costs across various therapy regimens over a 12-month period in the US and concluded that oral-based therapy regimens were generally associated with lower total costs and lower treatment burden [37]. Similar results were reported by Walter and colleagues who showed that the use of IRd is associated with cost savings compared to KRd, ERd, and DRd from the Austrian's payer's perspective [38]. Furthermore, oral administration of cancer drugs for example, avoids the inconvenience of infusions, the risk of infusion-related infections, and the need for additional administration visits [7, 8]. These aspects may account for discordance between the ability to stay on treatment observed in clinical trials and real-world practice. Hence, the ranges of duration of therapy in real-world reports were generally shorter than those reported in phase 3 clinical studies, with a larger gap seen with intravenous therapy regimens than oral-based therapy regimens [41]. Previous analyses showed that longer duration of therapy has been associated with prolonged PFS and OS [42, 43].

### Limitations of the study

The conducted study has several limitations. First, the economic modeling required assumptions that may limit interpretation of results. Second, to estimate average treatment duration with each triplet regimen within the model, PFS Kaplan–Meier data were modeled and extrapolated. Extrapolation was required for this analysis, because at the time of model building, median PFS had not yet been reached in the source trials. The same methodology for curve extrapolation was applied across all drugs and a scenario analysis with the second-best fit was performed to examine alternative plausible data inputs. Nonetheless, this approach did not allow for the use of indirect treatment comparisons nor correction of data based on some differences in trial populations. However, the evaluated trials specified similar inclusion and exclusion criteria resulting in similar populations across trials. Third, the treatment efficacy for each drug in each therapy line is assumed to be not correlated with the previous therapy line. It is likely that the efficacy of drugs differ by therapy line. However, to the best of our knowledge, no clinical trials have considered therapy lines beyond the third one. Finally, by focusing on the selected starting triplet therapies (IRd, KRd, ERd, DRd) for treating rrMM patients, the study might ignore further treatment options and may not necessarily reflect real-world treatment patterns. However, the consideration of three additional therapies (FVd, DVd, Pd) in third line is supposed to account for complex and varying treatment patterns among rrMM patients to some extent.

## Conclusion

The conducted BIA demonstrates that oral-based therapy regimens for rrMM offer cost advantages over intravenous-based therapy regimens. Saving effects were driven primarily by reduction of drug acquisition costs.
